# Establishment of a Flexible Real-Time Polymerase Chain Reaction-Based Platform for Detecting Prevalent Deafness Mutations Associated with Variable Degree of Sensorineural Hearing Loss in Koreans

**DOI:** 10.1371/journal.pone.0161756

**Published:** 2016-09-01

**Authors:** Kyu-Hee Han, Ah Reum Kim, Min Young Kim, Soyeon Ahn, Seung-Ha Oh, Ju Hun Song, Byung Yoon Choi

**Affiliations:** 1 Department of Otorhinolaryngology-Head and Neck Surgery, National Medical Center, Seoul, Korea; 2 Department of Otorhinolaryngology-Head and Neck Surgery, Seoul National University Bundang Hospital, Seoul National University College of Medicine, Seongnam, Korea; 3 Biomedical Research Institute, Seoul National University Hospital, Seoul, Korea; 4 Medical Research Collaborating Center, Seoul National University Bundang Hospital, Seongnam, Korea; 5 Department of Otorhinolaryngology-Head and Neck Surgery, Seoul National University Hospital, Seoul National University College of Medicine, Seoul, Korea; 6 Wide River Institute of Immunology, Seoul National University College of Medicine, Hongcheon, Korea; Hebrew University Hadassah Medical School, ISRAEL

## Abstract

Many cutting-edge technologies based on next-generation sequencing (NGS) have been employed to identify candidate variants responsible for sensorineural hearing loss (SNHL). However, these methods have limitations preventing their wide clinical use for primary screening, in that they remain costly and it is not always suitable to analyze massive amounts of data. Several different DNA chips have been developed for screening prevalent mutations at a lower cost. However, most of these platforms do not offer the flexibility to add or remove target mutations, thereby limiting their wider use in a field that requires frequent updates. Therefore, we aimed to establish a simpler and more flexible molecular diagnostic platform based on ethnicity-specific mutation spectrums of SNHL, which would enable bypassing unnecessary filtering steps in a substantial portion of cases. In addition, we expanded the screening platform to cover varying degrees of SNHL. With this aim, we selected 11 variants of 5 genes (*GJB2*, *SLC26A4*, *MTRNR1*, *TMPRSS3*, and *CDH23*) showing high prevalence with varying degrees in Koreans and developed the U-TOP™ HL Genotyping Kit, a real-time PCR-based method using the MeltingArray technique and peptide nucleic acid probes. The results of 271 DNA samples with wild type sequences or mutations in homo- or heterozygote form were compared between the U-TOP™ HL Genotyping Kit and Sanger sequencing. The positive and negative predictive values were 100%, and this method showed perfect agreement with Sanger sequencing, with a Kappa value of 1.00. The U-TOP™ HL Genotyping Kit showed excellent performance in detecting varying degrees and phenotypes of SNHL mutations in both homozygote and heterozygote forms, which are highly prevalent in the Korean population. This platform will serve as a useful and cost-effective first-line screening tool for varying degrees of genetic SNHL and facilitate genome-based personalized hearing rehabilitation for the Korean population.

## Introduction

Sensorineural hearing loss (SNHL) is one of the most common congenital diseases with an incidence of about 1 per 1,000 at birth and 1 per 300 by 4 years of age [1]. More than half of congenital SNHL cases are caused by genetic etiologies, and the non-syndromic and autosomal recessive form of SNHL is the most common [2].

Early detection of hearing loss is important for appropriate auditory rehabilitation. In this sense, genetic diagnosis can play a significant role by facilitating a correct diagnosis and prediction of auditory prognosis. Such information could help clinicians with making proper decisions regarding when and how to rehabilitate, recommending guidelines needed to prevent further aggravation and genetic counseling particularly in terms of the recurrent risk for SNHL.

Despite the advances of new sequencing technologies, there is still a need for a simpler screening technology with a more flexible platform as a first-line screening tool in clinical settings. Even with the generalization of various next-generation sequencing (NGS) platforms, the routine application of NGS for screening of hearing loss genes remains cost-prohibitive and complicated to analyze [3]. Several DNA chips targeting hereditary SNHL have been commercialized and offer clear advantages over NGS techniques, in terms of multiplexing [4]. However, once the chip is established, it is difficult to adjust target mutations. Novel ethnicity-specific alleles related to hearing loss are frequently discovered. In addition, a need exists for the ability to screen variants associated with varying degrees of SNHL because current screening methods have focused exclusively on pre-lingual, severe-to-profound hearing loss. Thus, it is important to develop a flexible screening kit that reflects ethnicity, can be easily updated to reflect sequence changes, and can cover a diverse degree of SNHL.

In this study, we employed MeltingArray technology (SeaSun Biomaterials, Daejeon, Korea), a peptide nucleic acid (PNA) probe-based fluorescence melting-curve analysis system that can be used in a conventional real-time PCR machine. Melting curve analysis using fluorescently labeled allele-specific PNA probes can enable rapid and reliable detection of DNA mutations including single nucleotide polymorphisms, insertions, and deletions [5]. PNA probes are dual-labeled, random-coiled, self-quenching probes. The probes comprise a short target-specific sequence, a fluorophore, and a dabsyl moiety attached at either end. In contrast to other types of molecular probes, PNA probes are not degraded by DNA polymerase during PCR elongation; thus, after PCR amplification, PNA probes can form stable duplexes with the target, selectively [6].

In this study, we aimed to develop a more efficient and adjustable genetic diagnostic kit using this MeltingArray technique as a first-line screening tool against prevalent mutations in Korean patients with varying degrees of SNHL.

## Materials and Methods

### Ethics statement

This study was approved by institutional review boards (IRBs) at Seoul National University Bundang Hospital (SNUBH; Seongnam, South Korea)(IRB-E-1501/282-303 and IRB-B-1007-105-402) and Seoul National University Hospital (SNUH; Seoul, South Korea) (IRBY-H-0905-041-281). We obtained written informed consent from all adult participants and from the parents or guardians of children participants.

### Selection of target SNHL mutations included in the diagnostic kit

We selected 11 variants from 5 genes (*GJB2*, *SLC26A4*, *MTRNR1*, *TMPRSS3*, and *CDH23*), which are found frequently in Koreans and cause varying degrees and phenotypes of SNHL, based on previous reports from several leading institutes in Korea as well as Japanese and Chinese studies [3, 7–23]. Mutations in *GJB2* and *SLC26A4*, which are the 2 most common deafness-related genes in Koreans, were chosen based on their frequency among Korean subjects with severe-to-profound SNHL [7–14, unpublished data generated at SNUBH] except p.V37I of *GJB2*. The p.V37I variant was selected owing to its being responsible up to 20% of postlingual mild-to-moderate hearing loss in the Korean population and 20% of postnatal, permanent childhood hearing loss in a Chinese population [13, 14]. The 1555A>G variant of mitochondrial 12S ribosomal RNA gene *(MTRNR1)*, the most common causative mutation of aminoglycoside-induced SNHL was chosen due to its potentially strong clinical implications, even though it shows a relatively low prevalence. This variant was found in 1.4% of the individuals comprising a Korean deaf population, while it appeared in approximately 3% of individuals among Chinese and Japanese populations [15–19]. Notably we also included the recently reported founder mutations, p.P240L of *CDH23* and p.A306T of *TMPRSS3* [20–23] (Table 1).

**Table 1:** Non-syndromic hearing loss genes and the related mutations detected with real-time PCR.

Gene	OMIM	Mutation	Wild type	Mutant type		Phenotypes	Frequency in Koreans	Frequency of each variant (among total variants from the gene)	Reference
				Homozygote	Heterozygote				
*GJB2 *	DFNB1A	p.V37I	TC**G**TT	TC**A**TT	TC**G/A**TT	postlingual mild to moderate hearing loss	16.9% (22/130) of arNSHL	9.8% (4/41)	Kim et al, 2015 [13]
		c.235delC*	CC**C**TG	CC-TG	CC**C/-**TG	wide range of hearing loss with low frequency residual hearing		39.0%(16/41)	
		c.299delAT	C**AT**GA	C—GA	C**AT/—**GA	prelingual severe to profound arNSHL			
		p.R143W	TC**C**GG	TC**T**GG	TC**C/T**GG	prelingual severe to profound arNSHL		26.8%(11/41)	
*SLC26A4 *	DFNB4	p.H723R*	CC**A**TG	CC**G**TG	CC**A/G**TG	pre- or perilingual onset fluctuating and progressive arNSHL with EVA, Pendred syndrome	6.5% (6/92) of recessive deaf [8]	40–61% (18/45-63/103) [9,12]	Park et al, 2003 and 2005 [8, 9], Rah et al, 2014 [12]
		c.IVS7-2A>G*	TC**A**GA	TC**G**GA	TC**A/G**GA			20–21% (9/45-22/103) [9,12]	
		p.T410M	CA**C**GG	CA**T**GG	CA**C/T**GG				
		p.L676Q	AC**T**GC	AC**A**GC	AC**T/A**GC				
*MTRNR1*		1555A>G	AG**A**CA	AG**G**CA	AG**A/G**CA	aminoglycoside-induced and NSHL, maternal transmission	1.4% (5/356, frequency of 1555A>G)		Bae et al, 2008 [16], Jeong et al, 2004 [17]
*TMPRSS3*	DFNB8/10	p.A306T	TC**G**CC	TC**A**CC	TC**G/A**CC	down sloping type or prelingual hearing loss with some residual hearing	5.9%(3/51) of arNSHL or 11.2%(3/27) of NSHL with low frequency residual hearing	50% (3/6)	Chung al, 2014 [20]
*CDH23*	DFNB12	p.P240L*	GC**C**TT	GC**T**TT	GC**C/T**TT	prelingual severe to profound arNSHL	3.1%(4/128) of pediatric severe to profound sporadic or arNSHL	85.7%(6/8)	Kim et al, 2015 [21]

* founder mutations in Korean

Bold and underlined letters indicate base pairs where the mutations occur.

arNSHL autosomal recessive nonsyndromic hearing loss

### Participants

We collected samples from patients with SNHL and their family members who visited the otolaryngology clinics of SNUH and SNUBH from May 2010 through May 2015 and who underwent genetic testing for causative mutations. From 854 participants, including 410 probands who were diagnosed with SNHL of varying degrees, we obtained 127 positive samples that had at least 1 of the 11 variants from the 5 genes in homozygous, single heterozygous, or compound heterozygous forms, as confirmed by Sanger sequencing. Accordingly, 144 normal control subjects who do not have any of 11 variants were also recruited. The calculation used to determine the normal control sample size required to validate our diagnostic kit with statistical significance is described below in the Statistical analysis section.

### Validation of the real-time PCR-based MeltingArray genetic diagnostic kit

To validate the performance of the molecular diagnostic kit using the MeltingArray technique, referred to as the U-TOP™ HL Genotyping Kit (SeaSun Biomaterials), we tested DNA samples collected in the SNUH and SNUBH and compared the results obtained using the U-TOP™ HL Genotyping kit with those obtained by traditional Sanger sequencing. All operators were provided with randomly ordered samples, which were anonymized by code numbers and were blinded in terms of previously reported genotypes.

Genomic DNA (gDNA) was isolated from whole blood samples using standard protocols (Gentra Puregene Blood Kit, Catalog No. 158389; Qiagen, Venlo, Netherlands), according to the manufacturer’s instructions. The stored gDNA samples had 260 nm/280 nm absorbance ratios of over 1.5, coded, and randomly ordered before being provided in a single-blinded manner to the operator for testing.

#### Real-time PCR

Real-time PCR was performed using the U-TOP™ HL Genotyping Kit (SeaSun Biomaterials) with a CFX96 Real-Time PCR Detection system (Bio-Rad, Hercules, CA, USA). Eleven mutations in 5 SNHL genes were examined using this kit according to the manufacturer’s manual. Briefly, 3 individual real-time PCR reactions were performed in 20-μl reactions containing 10 μl of 2× qPCR PreMix (SeaSun Biomaterials), 7 μl of primer and detection PNA probe mixture (HL set A, B, C), and 3 μL of DNA template (15 ng/μl) (Table 2). The reaction conditions for amplification and melting point analysis were 95°C for 10 min; 42 cycles of 95°C for 30 s, 58°C for 45 s, and 72°C for 45 s; followed by melting point analysis. Melting point analysis was performed using a denaturation step of 95°C for 5 min; touch-down, 1-min hybridization steps of 75°C, 55°C, and 45°C; and a stepwise temperature increase from 20 to 85°C at 1°C/step, with a 5 s interval between each step. The data were analyzed using Bio-Rad CFX manager v1.6 software (Bio-Rad). Mutations were characterized by the fluorescence signal of detection probes and corresponding melting temperatures (*T*_m_), according to the standard table provided by the kit manufacturer.

**Table 2:** List of sequences of primers and PNA probes used in real-time PCR.

Gene	Mutation	Direct-ion	Primer		PNA Probe	
			Primer Sequence (5' - 3')	Size (bp)	Probe Sequence (5' - 3')	Fluo-resce-nce
*GJB2*	p.V37I	F	GGGGTGTGAACAAACACTCCA	161	CCACAACGAGGATC	FAM
		R	ATCTCCCCACACCTCCTTTGC			
	c.235delC	F	ACGATCACTACTTCCCCATCTCC	145	CAGGGCCCATAGCCG	Cy5
	c.299delAT	R	CTCTTTATCTCCCCCTTGATGAAC		CATGTCTCCGGTA	HEX
	p.R143W	F	GGTGGACCTACACAAGCAGCA	90	GATGACCCGGAAGAA	Texas Red
		R	TGGAGAAGCCGTCGTACATGA			
*SLC16A4*	p.T410M	F	TTTGGGATCAGCAACATCTTCTC	154	TCCCGCACGGCCGTC	FAM
		R	CCATTCCTCGACTTGTTCTCTGA		CCGCATGG	FAM
	p.L676Q	F	CAATCCATAGCCTTGTGCTTGAC	151	AGATCACTGCGGG	HEX
		R	TTGCAATACTGGACAACCCACAT			
	p.H723R	F	AGCCTGGGCAATAGAATGAGACT	229	GGTCCATGATGCTA	Texas Red
		R	AAATGGAACCTTGACCCTCTTGA			
	c.IVS7-2A>G	F	CACAAAATCCCAGTCCCTATTCC	221	TTTTATTTCAGACGATAA	Cy5
		R	CCCTTGGGATGGATTTAACAATG			
*MTRNR1*	1555A>G	F	GGTCGAAGGTGGATTTAGCAG	202	ACGACTTGTCTCCTCTA	HEX
		R	GCTACACTCTGGTTCGTCCAA			
*TMPRSS3*	p.A306T	F	ATTTCAGCTTGTACCTCCCCAAG	189	ATGACATCGCCCTTATG	Cy5
		R	ACCCAGATGTACCATTGAACGTG			
*CDH23*	p.P240L	F	ACTTGGCCATCATCATCACAGAT	162	AACCTGCCTTACAGC	Texas Red
		R	CCCTAACAGGAGCTCAGAAGGAA			

F: forward direction, R: reverse direction

#### Sanger sequencing

Sanger sequencing was performed as follows. A total of 8 sets of primers indicated in Table 3 were used to amplify gDNA regions containing the mutations. Polymerase chain reaction (PCR) was performed in 20-μl reactions containing 3 μL purified gDNA (15 ng/μl), 10 μl 2× qPCR PreMix (SeaSun Biomaterials), and 0.5 μl each of a forward and reverse primer (10 mM). Subsequently, thermocycling was performed using 40 cycles at 95°C for 30 s, 58°C for 45 s, 72°C for 45 s, with a final elongation step performed at 72°C for 5 min. The products were electrophoresed on a 2% agarose gel and visualized with ethidium bromide staining under ultraviolet light to verify their sizes and assess their quantities. PCR products were sequenced using the ABI BigDye Terminator v3.1 Cycle Sequencing Kit (Applied Biosystems, Waltham, MA, USA), according to the manufacturer’s instructions. The sequencing primers were the same as the PCR primers. Sequencing reaction products were electrophoresed on an ABI 3500XL genetic analyzer (Applied Biosystems). Sequence data were analyzed using an ABI 3500XL DNA Analyzer (Applied Biosystems). All gDNA samples were examined individually with each of the 8 sets of primers in both directions.

**Table 3:** List of primer sequences used in Sanger sequencing.

Gene	Mutation	Direction	Primer	
			Primer Sequence (5' - 3')	Size (bp)
*GJB2*	p.V37I, c.235delC, c.299delAT, p.R143W	F	GGGGTGTGAACAAACACTCCA	456
		R	TGGAGAAGCCGTCGTACATGA	
*SLC16A4*	p.T410M	F	GGATCAGCAACATCTTCTCAGGA	158
		R	CTCTGTTGCCATTCCTCGACTT	
	p.L676Q	F	CAATCCATAGCCTTGTGCTTGAC	151
		R	TTGCAATACTGGACAACCCACAT	
	p.H723R	F	AGCCTGGGCAATAGAATGAGACT	229
		R	AAATGGAACCTTGACCCTCTTGA	
	c.IVS7-2A>G	F	CACAAAATCCCAGTCCCTATTCC	221
		R	CCCTTGGGATGGATTTAACAATG	
*MTRNR1*	1555A>G	F	GGTCGAAGGTGGATTTAGCAG	202
		R	GCTACACTCTGGTTCGTCCAA	
*TMPRSS3*	p.A306T	F	ATTTCAGCTTGTACCTCCCCAAG	189
		R	ACCCAGATGTACCATTGAACGTG	
*CDH23*	p.P240L	F	ACTTGGCCATCATCATCACAGAT	162
		R	CCCTAACAGGAGCTCAGAAGGAA	

F: forward direction, R: reverse direction

### Statistical analysis

To evaluate the performance of the U-TOP™ HL Genotyping Kit, the positive predictive value (PPV), negative predictive value (NPV), and 95% confidence intervals (CI) for the PPV and NPV were calculated.

The 1-sample, non-inferiority test was used at a significance level of 0.05 and a statistical power of 80%. The average of PPV and NPV were obtained from 12 reference articles where methods using matrix-assisted laser desorption ionization-time-of-flight mass spectrometry or real-time PCR were compared with Sanger sequencing in detecting mutations of GJB2 or other human genes. The average PPV and NPV were 96.7% and 92.5%, respectively, and the differences from the lower margin of the 95% CI (δ, the non-inferiority margin) were -4.7% and -6.5% respectively [24–35]. Based on the reference articles, the sizes of positive and normal samples were calculated using the equation below, considering a 10% dropout rate.

$$n=\frac{{\left(Z_{\alpha /2}+Z_{\beta }\right)}^{2}p(1-p)}{{\left(\delta -\left|p-p_{0}\right|\right)}^{2}}$$

*p* = the average PPV and NPV values from the reference articles

*p*_0_ = the expected PPV and NPV for this study (equivalence)

*Z*_*α*/2_ = 1.96

*Z*_*β*_ = 0.842

In addition, to assess the agreement between 2 tests, we calculated the kappa (κ) statistic, which is widely considered to reflect almost perfect agreement when the kappa value is between 0.81 and 1.00 [36].

## Results

We tested 271 gDNA specimens (127 samples with mutations and 144 control samples) using both the U-TOP™ HL Genotyping Kit and Sanger sequencing. The U-TOP HL Genotyping Kit showed distinct melting curves specific to each allele, with *T*_m_ values for each diagnostic melting peak detected exactly as predicted. Sanger sequencing enabled detection of 153 mutations from 127 samples, and these results were replicated using the U-TOP™ HL Genotyping Kit (S1 Table and Table 4). The 153 mutations identified consisted of 135 heterozygous and 18 homozygous mutations. The p.H723R variant of *SLC26A4* was the most common, and c.235delC of *GJB2* was the most commonly detected among samples with a homozygous mutation (Table 5). Among 127 positive samples, 18 samples carried homozygous target variants while 88 samples carried one or more single heterozygous target variants. The other 21 samples were compound heterozygotes of one of the five target genes. Two or more different target variants were detected in 25 samples and 21 were compound heterozygotes of either *GJB2* or *SLC26A4* (Table 6).

**Table 4:** Test results of the U-TOP™ HL Genotyping Kit and Sanger sequencing.

			Sanger sequencing												
			*GJB2*				*SLC26A4*				*MTRNR1*	*TMPRSS3*	*CDH23*	Wild type	Total
			p.V37I	c.299delAT	c.235delC	p.R143W	p.T410M	p.L676Q	p.H723R	c.IVS7-2A>G	1555A>G	p.A306T	p.P240L		
**Real-time PCR**	*GJB2*	p.V37I	15	0	0	0	0	0	0	0	0	0	0	0	15
		c.299delAT	0	10	0	0	0	0	0	0	0	0	0	0	10
		c.235delC	0	0	24	0	0	0	0	0	0	0	0	0	24
		p.R143W	0	0	0	12	0	0	0	0	0	0	0	0	12
	*SLC26A4*	p.T410M	0	0	0	0	8	0	0	0	0	0	0	0	8
		p.L676Q	0	0	0	0	0	7	0	0	0	0	0	0	7
		p.H723R	0	0	0	0	0	0	35	0	0	0	0	0	35
		c.IVS7-2A>G	0	0	0	0	0	0	0	18	0	0	0	0	18
	*MTRNR1*	1555A>G	0	0	0	0	0	0	0	0	4	0	0	0	4
	*TMPRSS3*	p.A306T	0	0	0	0	0	0	0	0	0	8	0	0	8
	*CDH23*	p.P240L	0	0	0	0	0	0	0	0	0	0	12	0	12
	Wild type		0	0	0	0	0	0	0	0	0	0	0	144	144
	Total		15	10	24	12	8	7	35	18	4	8	12	144	297

**Table 5:** Distribution of mutations detected by the U-TOP™ HL Genotyping Kit, according to the variants and genotypes.

Genotype	*GJB2*				*SLC26A4*				*MTRNR1*	*TMPRSS3*	*CDH23*	Total
	p.V37I	c.299delAT	c.235delC	p.R143W	p.T410M	p.L676Q	p.H723R	c.IVS7-2A>G	1555A>G	p.A306T	p.P240L	
Heterozygote	14	10	19	12	8	7	32	15	0	8	10	135
Homozygote	1	0	5	0	0	0	3	3	4	0	2	18
Total	15	10	24	12	8	7	35	18	4	8	12	153

**Table 6:** Genotyping results of subjects with two or more different mutations.

Gene	Mutation		Patients ID	No of specimen
	Gene 1	Gene 2		
*SLC26A4+SLC26A4*	*SLC26A4*:p.H723R/c.IVS7-2A>G		SB104-196, SB129-222, SNUH192-432-J154, SNUH33-74, SB214-419	15
	*SLC26A4*:p.L676Q/p.H723R		SB16-35, SB28-61, SNUH154-334, SB16-34	
	*SLC26A4*:p.T410M/p.H723R		SNUH129-267, SNUH133-276, SNUH179-398, SB23-55, SB221-432	
* *	*SLC26A4*:p.T410M/c.IVS7-2A>G		SB23-54	
*GJB2+GJB2*	*GJB2*:c.235delC/p.R143W		SNUH70-160, SNUH185-419-J152	5
	*GJB2*:p.V37I/p.R143W		SNUH95-209, SNUH95-256	
	*GJB2*:p.V37I/c.235delC		SNUH42-94	
*GJB2+SLC26A4*	*GJB2*:p.V37I (het)	*SLC26A4*:c.IVS7-2A>G (het)	SNUH162-356	3
	*GJB2*:p.V37I (het)	*SLC26A4*:c.IVS7-2A>G (homo)	SNUH162-355	
	*GJB2*:c.235delC (het)	*SLC26A4*:p.H723R (het)	SNUH35-76	
*GJB2+MTRNR1*	*GJB2*:p.R143W (het)	*MTRNR1*:1555A>G (homo)	SNUH60-139	1
*GJB2+GJB2+SLC26A4*	*GJB2*:c.299delAT/c.235delC	*SLC26A4*:p.H723R (het)	SNUH35-75	1
			**Total**	25

Het: single heterozygote, Homo: homozygote

**Fig 1:**
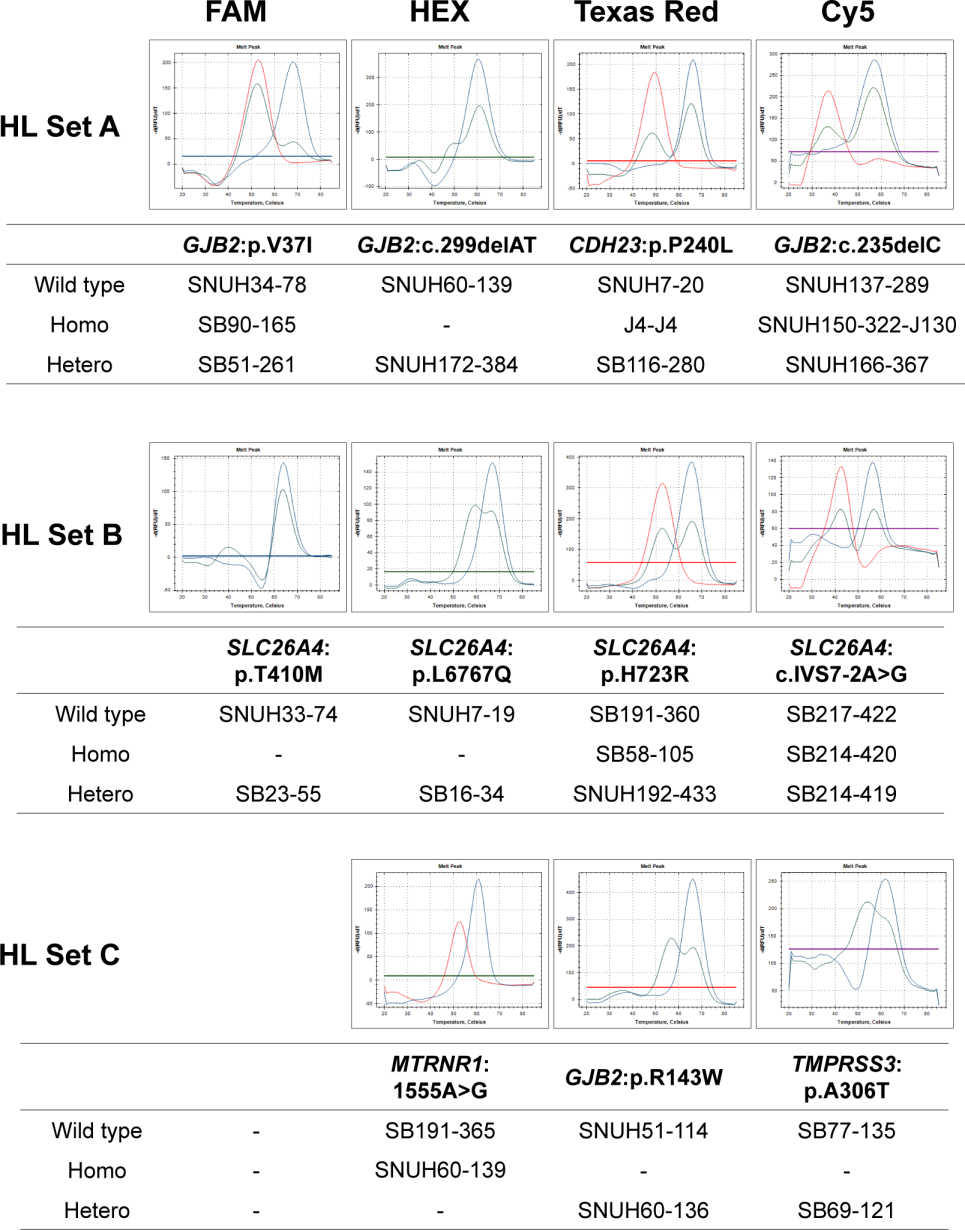
Representing melting peaks of eleven variants. Lines colored in red, green, and blue indicate homozygous mutant, heterozygous mutant, and wild type sequences, respectively for eleven variants. Each HL set occupied 1 well and contained 3 or 4 PNA probes labeled with fluorophores for detection in the FAM, HEX, Texas Red, and Cy5 channels.

Fig 1 shows representative melting peaks for each variant in a homozygous and heterozygous state, as well as those of the wild type sequences. In addition to heterozygous mutations, homozygous mutations were clearly detectable with a single melting peak at *T*_m_ that was distinct from that of the wild type sequence.

The PPV was 100.0% (95% CI: 97.1%–100.0%) and the NPV was 100.0% (95% CI: 97.4%–100.0%), without any false negative or false positive results. The differences in the PPV and NPV from the lower margin of the 95% CI were 2.9% and 2.6%, respectively, and these were lower than predetermined non-inferiority margins from the references (4.7% and 6.5%, respectively). Thus, we can conclude that performance of the U-TOP™ HL Genotyping Kit was similar to Sanger sequencing. The lower margins of the 95% CI of the PPV and NPV were 97.1% and 97.4%, respectively, and these were higher than 95.3% and 93.5%. These data confirmed that the PPV and NPV of U-TOP™ HL Genotyping Kit were comparable to those from the references.

The Kappa value was 1.00, as calculated using the equation below. Therefore, the results with U-TOP™ HL Genotyping Kit showed perfect agreement with the Sanger sequencing results (Table 7).

$$k=\frac{2(ad-bc)}{\left(a+b)(b+d)+(a+c)(c+d\right)}=\frac{2(127\times 144)-(0\times 0)}{\left(127+0)(0+144)+(127+0)(0+144\right)}=1.00$$

**Table 7:** Comparison of mutation-detection results obtained with the U-TOP™ HL Genotyping Kit and Sanger sequencing.

		Sanger sequencing		Total
		Positive	Negative	
Real-time PCR kit(U-TOP™ HL Genotyping Kit)	Positive	127 (a)	0 (b)	127
	Negative	0 (c)	144 (d)	144
Total		127	144	271

The detection of 1 or more mutations was scored as a positive result, and wild type sequences were scored as negative.

To predict a detection rate of this kit among various degrees and types of SNHL, we employed our entire SNHL cohort with such a distribution. We counted the number of probands who had at least one of 11 variants from 5 genes from 410 probands of our entire SNHL cohort to predict the degree to which these selected variants would account for a diverse spectrum of SNHL in a Korean ethnicity. Among the 410 probands, 83 had one or more of the 11 variants studied, with a detection rate of 20.2%.

To extrapolate the predicted detection rate for prelingual SNHL by this kit from our data, we counted the number of subjects with prelingual SNHL and also the number of subjects carrying any of the nine target variants of the *GJB2*, *SLC26A4* and *CDH23* genes selected for our U-TOP™ HL Genotyping Kit. A total of 218 prelingual SNHL subjects were included in our SNHL cohort, and 35.8% (78/218) of these cases were caused by the nine target variants in this kit. Furthermore, these nine target variants accounted for 89.7% (78/87) of 87 prelingual SNHL caused by these 3 genes, showing a significant usefulness of this kit to easily screen prelingual SNHL cases.

## Discussion

Early diagnosis and auditory rehabilitation of hearing loss in children is very important; however, it is not always feasible, especially in cases of autosomal recessive SNHL. A child with hearing impairment would be unexpected for such parents because they and their relatives usually have normal hearing and in most cases, the child would not be comorbid with other abnormal phenotypes. An unanticipated hearing deficiency might result in delayed detection and intervention against hearing loss in children, thereby leading to poorer outcomes if the critical period of language development is missed. The genetic diagnosis of hearing loss helps clinicians and patients to delineate the characteristics of hearing loss with greater clarity. In this study, we sought to facilitate accurate diagnosis of genetic hearing loss by establishing a more convenient and time-efficient genetic diagnostic kit in reference to known genetic information for the Korean population.

In terms of efficiency, the U-TOP™ HL Genotyping Kit offers advantages over other methods, such as direct sequencing and DNA chips. Firstly, this kit utilizes the conventional real-time PCR machine that is commonly used in many laboratories. Additional expensive equipment is not necessary. Mutations in hearing loss-associated genes are distinguished in 1 well with 3 or 4 different fluorescent dyes, as well as by the shapes of melting peaks that appear at specific melting temperatures to differentiate the genotypes. Accordingly, the analysis of 11 variants requires only 3 wells, with 3 or 4 mutations assayed per well. Therefore, this kit offers the merits of high throughput and multiplexing with a small amount of sample. Moreover, the use of real-time PCR and PNA probes have advantages in terms of flexibility over DNA chips, which have been mainly developed as screening tools, because we can easily alter the targeted mutations by adding or removing PNA probes and primers. With increasing attention being paid to genetic diagnosis and advancements in related technologies, new causative SNHL mutations are being discovered, and their inferred clinical significance in primary screening is constantly changing. The kit developed in this study is expected to fulfill the need for a low-cost screening method with enhanced flexibility and efficiency.

Recently, Sagong et al. reported the development of a screening method for hearing loss mutations using multiplex SNaPshot minisequencing, which is also rapid and offers easy data analysis [37]. This method also requires relatively universal equipment and comprises short steps: a thermocycler is used for PCR and single base extension reactions, and a DNA sequencer is used for electrophoresis and analysis. In addition, similar to real-time PCR, independent amplification with specific primers for target variants allows researchers to easily incorporate other mutations of interest. However, the U-TOP™ HL Genotyping Kit still has a critical advantage over multiplex SNaPshot minisequencing in that the U-TOP™ HL Genotyping Kit requires only a real-time PCR machine. In addition, the risk of sample contamination and sample loss is much lower with the kit than other methods, as the assay is performed in a single step in closed wells during the reaction and analysis steps. MassARRAY system combined iPLEX assay is also a high-throughput genotyping technology using MALDI-TOF mass spectrometry [38]. This MassARRAY system can handle large number of variants in multiple samples simultaneously at relatively low genotyping cost per patient when it once setup. However MassARRAY system is still very costly to be widely used because it depends on very expensive platform for the analysis, which is not usually equipped. In contrast, our system needs only real-time PCR machine universally used in many laboratories. We can start up genetic diagnosis with much lower initial purchasing cost or utilizing existing PCR machine. Moreover, our system is also a flexible platform where we can expand the number of variants by designing new PNA probes against additional variants. MassARRAY system may have advantage in screening a large number of mutations scattered over multiple genes in the ethnic groups with extreme genetic heterogeneity of SNHL. However, in the East Asian groups including Korean population, which have distinct hot spots or founder mutations in several genes, it may be a more practical strategy to screen out such mutations with easily accessible system. In this sense, we included five hotspot or founder mutations over four genes in East Asian including Koreans in our kit (Table 1).

**Fig 2:**
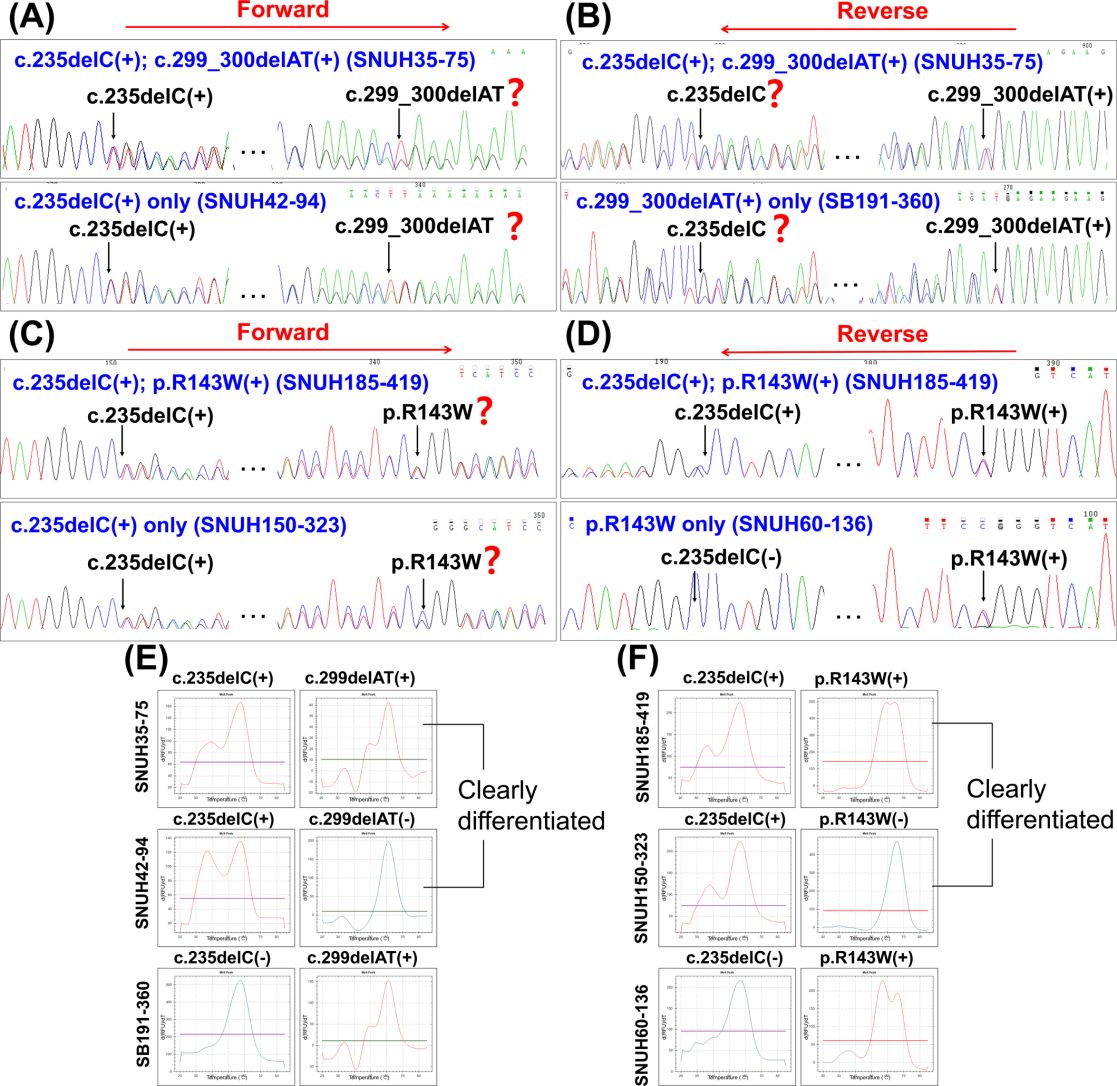
Sequence chromatograms of compound heterozygous mutations, including frameshift mutations within a single amplicon. (A, B) Comparison of the sequence chromatograms of a compound heterozygous *GJB2* c.235delC and c.299_300delAT mutation (upper) with those of the carrier of the each variant (bottom), using a forward primer (A) or a reverse primer (B), respectively: Both deletion mutations cause a frameshift, and it is easy to miss these mutations located downstream of a deletion mutant with either forward or reverse sequencing only. (C, D) The sequence chromatograms of a compound heterozygous *GJB2* c.235delC and p.R143W mutation (upper) were compared with those of carrier of each variant (bottom), using a forward primer (C) and a reverse primer (D). Detection of a missense mutation, p.R143W, downstream to c.235delC can be detected by reverse sequencing, but not only by forward sequencing only if the two mutations are in a single amplicon. (E, F) Corresponding melting peaks of the compound heterozygous mutations of *GJB2* and the carrier of the each variant. Curves show double peaks of heterozygote in red and single peak of wild type in blue at its specific melting temperatures. All mutations can be easily discernible.

More importantly, the use of melting peak analysis makes the interpretation of results much easier, intuitive, and more accurate than the use of Sanger sequencing in specific situations. In the case of an amplicon that has 2 or more mutations including frameshift mutation, unidirectional Sanger sequencing, either in a forward or reverse direction, may have a limitation detecting mutations occurring downstream of a frameshift mutation by aligning the sample trace to reference trace, as illustrated in Fig 2. However, the U-TOP™ HL Genotyping Kit could be used to detect this type of compound heterozygous mutations more accurately and much more easily because the MeltingArray technique uses a specific melting temperature of a PNA probe against each target mutations (Fig 2). This advantage makes this screening kit more useful for the Korean population because these variants that can be amplified in a single amplicon are found in high prevalence. For example, we encountered 4 (7.41%) of such cases among 54 samples with GJB2 mutations in our cohort.

The variants currently tested with the U-TOP™ HL Genotyping Kit have meaningful clinical implications. Prelingual severe-to-profound SNHL subjects who carry p.P240L of *CDH23* are good candidates for early cochlear implantation. The p.P240L variant has been reported to be a predominant *CDH23* mutation among Japanese and Korean people, accounting for around 45% or even over 50% of the total number of *CDH23* mutations and has recently been reported to cause a strong founder effect in Koreans by Kim *et al*. [21–23, 39]. Thus, screening p.P240L in *CDH23* from non-*GJB2* and non-enlarged vestibular aqueduct subjects with autosomal recessive prelingual hearing loss would offer a cost-effective diagnostic yield. Considering that *GJB2*, *SLC26A4*, and *CDH23* are the 3 most common causative genes of prelingual SNHL in Koreans [40] and that the founder alleles of these 3 genes are all included, our kit alone is expected to cover a substantial portion of prelingual severe-to-profound genetic SNHL in Koreans. The 9 mutations of 3 genes included in this kit can cover 35.8% of prelingual SNHL among our entire SNHL cohort with varying degrees of SNHL. Furthermore, out kit is expected to account for upto 90% of prelingual SNHL cases caused by the main three prelingual SNHL causative genes in Koreans and East Asians, without Sanger sequencing.

This high diagnostic yield of this genotyping kit is not necessarily limited to prelingual severe-to-profound SNHL cases. Mutations of the *GJB2* gene are commonly, but not always related with a severe-to-profound degree of hearing loss with autosomal recessive inheritance. The variant p.V37I is usually responsible for mild-to-moderate and later-onset hearing loss and its carrier frequency is very high (up to 18.2%) among Korean with mild or slight hearing loss [3, 13, 41]. Moreover, the p.A306T mutation of *TMPRSS3*, which has been reported to be a founder allele in Koreans, can be associated with the postlingual ski slope type of progressive SNHL [20]. Detection of p.A306T in *TMPRSS3* using this kit can potentially provide surgeons with valuable information regarding how and when to rehabilitate SNHL, as the rapidity of SNHL progression related to *TMPRSS3* is likely to depend on the type of the mutant allele of *TMPRSS3* in a *trans* configuration with p.A306T [20, 42]. The residual low frequency hearing would aggravate very rapidly or be maintained for scores of years, depending on the combination of the 2 mutant alleles. With advances in various hearing rehabilitation devices such as combined electric and acoustic stimulation and techniques to preserve residual hearing in low frequencies during cochlear implantation surgery, it became increasingly important to prognose the hearing status with greater accuracy to decide when and how to rehabilitate hearing-impaired patients [43], and clarification of the genetic etiology can help in this regard.

By including prevalent mutations associated with moderate SNHL or postlingual ski slope SNHL, the kit accounted for 20% of the entire SNHL cohort comprising varying degrees of SNHL. Considering that our kit was predicted to cover 35.8% of prelingual SNHL cases and that about 50% of prelingual SNHL cases in general are thought to have a genetic etiology, this kit might be expected to possibly cover upto 70% of prelingual cases in Koreans. However it is also possible that our SNHL cohort might have a recruitment bias toward more genetic cases, which may have exaggerated out predicted detection rate of this kit for prelingual SNHL cases. Nevertheless, almost 90% of subjects with variants somewhere in the main three genes for prelingual SNHL genes (*GJB2*, *SLC26A4*, and *CDH23*) can be expected to be detected by this kit in Koreans. For the next step for the subjects with no detected mutations by this kit, we routinely apply targeted panel sequencing of known deafness genes as described [40, 44, 45]. Whole exome sequencing is performed for the unresolved cases even with targeted panel sequencing.

To our knowledge, this is the first screening kit that can cover a significant portion of more general population with a broad spectrum of hearing loss, rather than a biased cohort with only severe to profound hearing loss. Therefore, screening for mutations with the U-TOP™ HL Genotyping Kit targeting a wide range of hearing loss as well as prelingual severe-to-profound SNHL is a very useful and efficient approach for clinicians and researchers to begin genetic diagnosis.

## Conclusion

We developed a new kit for genetic hearing loss and demonstrated excellent results in detecting hearing loss-related genetic mutations prevalent in Korean population in homo- and heterozygote forms, compared with Sanger sequencing. This kit is an efficient and flexible tool that can be used to detect mutations causing varying degrees and phenotypes of hearing loss. This kit is expected to serve as a primary screening tool for genetic hearing loss and could be helpful in genome-based and personalized hearing rehabilitation.

## Supporting Information

S1 Table Genotyping results of 127 positive samples by the U-TOP™ HL Genotyping Kit (DOCX)
