# Supplementary material for: Establishment of a Flexible Real-Time Polymerase Chain Reaction-Based Platform for Detecting Prevalent Deafness Mutations Associated with Variable Degree of Sensorineural Hearing Loss in Koreans
Source: PLoS One. 2016 Sep 1;11(9):e0161756. doi: 10.1371/journal.pone.0161756 (PMC5008798; doi:10.1371/journal.pone.0161756)
Supplement: S1 Table — (DOCX) [file pone.0161756.s001.docx]

**S1 Table. Genotyping results of 127 positive samples by the U-TOP™ HL Genotyping Kit**

| **Patient number** | **Mutation 1** | | **Mutation 2** | | **Mutation 3** | |
| --- | --- | --- | --- | --- | --- | --- |
| 1 | *GJB2*:235delC | Heterozygote |  |  |  |  |
| 2 | *GJB2*:235delC | Heterozygote |  |  |  |  |
| 3 | *GJB2*:235delC | Heterozygote | *GJB2*:R143W | Heterozygote |  |  |
| 4 | *GJB2*:235delC | Homozygote |  |  |  |  |
| 5 | *GJB2*:235delC | Heterozygote | SLC26A4:H723R | Heterozygote |  |  |
| 6 | *GJB2*:235delC | Heterozygote |  |  |  |  |
| 7 | *GJB2*:235delC | Homozygote |  |  |  |  |
| 8 | *GJB2*:235delC | Heterozygote |  |  |  |  |
| 9 | *GJB2*:235delC | Homozygote |  |  |  |  |
| 10 | *GJB2*:235delC | Homozygote |  |  |  |  |
| 11 | *GJB2*:235delC | Homozygote |  |  |  |  |
| 12 | *GJB2*:235delC | Heterozygote |  |  |  |  |
| 13 | *GJB2*:235delC | Heterozygote |  |  |  |  |
| 14 | *GJB2*:235delC | Heterozygote |  |  |  |  |
| 15 | *GJB2*:235delC | Heterozygote | *GJB2*:R143W | Heterozygote |  |  |
| 16 | *GJB2*:235delC | Heterozygote |  |  |  |  |
| 17 | *GJB2*:235delC | Heterozygote |  |  |  |  |
| 18 | *GJB2*:235delC | Heterozygote |  |  |  |  |
| 19 | *GJB2*:235delC | Heterozygote |  |  |  |  |
| 20 | *GJB2*:235delC | Heterozygote |  |  |  |  |
| 21 | *GJB2*:235delC | Heterozygote |  |  |  |  |
| 22 | *GJB2*:235delC | Heterozygote |  |  |  |  |
| 23 | *GJB2*:299delAT | Heterozygote |  |  |  |  |
| 24 | *GJB2*:299delAT | Heterozygote |  |  |  |  |
| 25 | *GJB2*:299delAT | Heterozygote |  |  |  |  |
| 26 | *GJB2*:299delAT | Heterozygote |  |  |  |  |
| 27 | *GJB2*:299delAT | Heterozygote |  |  |  |  |
| 28 | *GJB2*:299delAT | Heterozygote | *GJB2*:235delC | Heterozygote | SLC26A4:H723R | Heterozygote |
| 29 | *GJB2*:299delAT | Heterozygote |  |  |  |  |
| 30 | *GJB2*:299delAT | Heterozygote |  |  |  |  |
| 31 | *GJB2*:299delAT | Heterozygote |  |  |  |  |
| 32 | *GJB2*:299delAT | Heterozygote |  |  |  |  |
| 33 | *GJB2*:R143W | Heterozygote |  |  |  |  |
| 34 | *GJB2*:R143W | Heterozygote |  |  |  |  |
| 35 | *GJB2*:R143W | Heterozygote |  |  |  |  |
| 36 | *GJB2*:R143W | Heterozygote |  |  |  |  |
| 37 | *GJB2*:R143W | Heterozygote |  |  |  |  |
| 38 | *GJB2*:R143W | Heterozygote |  |  |  |  |
| 39 | *GJB2*:R143W | Heterozygote |  |  |  |  |
| 40 | *GJB2*:V37I | Heterozygote |  |  |  |  |
| 41 | *GJB2*:V37I | Heterozygote |  |  |  |  |
| 42 | *GJB2*:V37I | Heterozygote | *GJB2*:R143W | Heterozygote |  |  |
| 43 | *GJB2*:V37I | Heterozygote | *GJB2*:R143W | Heterozygote |  |  |
| 44 | *GJB2*:V37I | Homozygote |  |  |  |  |
| 45 | *GJB2*:V37I | Heterozygote |  |  |  |  |
| 46 | *GJB2*:V37I | Heterozygote | SLC26A4:IVS7-2A>G | Heterozygote |  |  |
| 47 | *GJB2*:V37I | Heterozygote | *GJB2*:235delC | Heterozygote |  |  |
| 48 | *GJB2*:V37I | Heterozygote |  |  |  |  |
| 49 | *GJB2*:V37I | Heterozygote |  |  |  |  |
| 50 | *GJB2*:V37I | Heterozygote |  |  |  |  |
| 51 | *GJB2*:V37I | Heterozygote |  |  |  |  |
| 52 | *GJB2*:V37I | Heterozygote |  |  |  |  |
| 53 | *GJB2*:V37I | Heterozygote |  |  |  |  |
| 54 | *SLC26A4*:H723R | Heterozygote | *SLC26A4*:IVS7-2A>G | Heterozygote |  |  |
| 55 | *SLC26A4*:H723R | Homozygote |  |  |  |  |
| 56 | *SLC26A4*:H723R | Heterozygote |  |  |  |  |
| 57 | *SLC26A4*:H723R | Homozygote |  |  |  |  |
| 58 | *SLC26A4*:H723R | Heterozygote | *SLC26A4*:IVS7-2A>G | Heterozygote |  |  |
| 59 | *SLC26A4*:H723R | Heterozygote |  |  |  |  |
| 60 | *SLC26A4*:H723R | Heterozygote | *SLC26A4*:IVS7-2A>G | Heterozygote |  |  |
| 61 | *SLC26A4*:H723R | Heterozygote | *SLC26A4*:IVS7-2A>G | Heterozygote |  |  |
| 62 | *SLC26A4*:H723R | Heterozygote |  |  |  |  |
| 63 | *SLC26A4*:H723R | Heterozygote |  |  |  |  |
| 64 | *SLC26A4*:H723R | Heterozygote |  |  |  |  |
| 65 | *SLC26A4*:H723R | Heterozygote |  |  |  |  |
| 66 | *SLC26A4*:H723R | Heterozygote |  |  |  |  |
| 67 | *SLC26A4*:H723R | Heterozygote |  |  |  |  |
| 68 | *SLC26A4*:H723R | Heterozygote |  |  |  |  |
| 69 | *SLC26A4*:H723R | Heterozygote |  |  |  |  |
| 70 | *SLC26A4*:H723R | Heterozygote |  |  |  |  |
| 71 | *SLC26A4*:H723R | Homozygote |  |  |  |  |
| 72 | *SLC26A4*:H723R | Heterozygote |  |  |  |  |
| 73 | *SLC26A4*:H723R | Heterozygote |  |  |  |  |
| 74 | *SLC26A4*:H723R | Heterozygote |  |  |  |  |
| 75 | *SLC26A4*:H723R | Heterozygote | *SLC26A4*:IVS7-2A>G | Heterozygote |  |  |
| 76 | *SLC26A4*:H723R | Heterozygote |  |  |  |  |
| 77 | *SLC26A4*:H723R | Heterozygote |  |  |  |  |
| 78 | *SLC26A4*:IVS7-2A>G | Homozygote | GJB2:V37I | Heterozygote |  |  |
| 79 | *SLC26A4*:IVS7-2A>G | Heterozygote |  |  |  |  |
| 80 | *SLC26A4*:IVS7-2A>G | Heterozygote |  |  |  |  |
| 81 | *SLC26A4*:IVS7-2A>G | Heterozygote |  |  |  |  |
| 82 | *SLC26A4*:IVS7-2A>G | Heterozygote |  |  |  |  |
| 83 | *SLC26A4*:IVS7-2A>G | Heterozygote |  |  |  |  |
| 84 | *SLC26A4*:IVS7-2A>G | Heterozygote |  |  |  |  |
| 85 | *SLC26A4*:IVS7-2A>G | Heterozygote |  |  |  |  |
| 86 | *SLC26A4*:IVS7-2A>G | Heterozygote |  |  |  |  |
| 87 | *SLC26A4*:IVS7-2A>G | Homozygote |  |  |  |  |
| 88 | *SLC26A4*:IVS7-2A>G | Homozygote |  |  |  |  |
| 89 | *SLC26A4*:L676Q | Heterozygote | *SLC26A4*:H723R | Heterozygote |  |  |
| 90 | *SLC26A4*:L676Q | Heterozygote |  |  |  |  |
| 91 | *SLC26A4*:L676Q | Heterozygote | *SLC26A4*:H723R | Heterozygote |  |  |
| 92 | *SLC26A4*:L676Q | Heterozygote | *SLC26A4*:H723R | Heterozygote |  |  |
| 93 | *SLC26A4*:L676Q | Heterozygote | *SLC26A4*:H723R | Heterozygote |  |  |
| 94 | *SLC26A4*:L676Q | Heterozygote |  |  |  |  |
| 95 | *SLC26A4*:L676Q | Heterozygote |  |  |  |  |
| 96 | *SLC26A4*:T410M | Heterozygote | *SLC26A4*:IVS7-2A>G | Heterozygote |  |  |
| 97 | *SLC26A4*:T410M | Heterozygote | *SLC26A4*:H723R | Heterozygote |  |  |
| 98 | *SLC26A4*:T410M | Heterozygote | *SLC26A4*:H723R | Heterozygote |  |  |
| 99 | *SLC26A4*:T410M | Heterozygote | *SLC26A4*:H723R | Heterozygote |  |  |
| 100 | *SLC26A4*:T410M | Heterozygote | *SLC26A4*:H723R | Heterozygote |  |  |
| 101 | *SLC26A4*:T410M | Heterozygote |  |  |  |  |
| 102 | *SLC26A4*:T410M | Heterozygote | *SLC26A4*:H723R | Heterozygote |  |  |
| 103 | *SLC26A4*:T410M | Heterozygote |  |  |  |  |
| 104 | *MTRNR1*:1555A>G | Homozygote | GJB2:R143W | Heterozygote |  |  |
| 105 | *MTRNR1*:1555A>G | Homozygote |  |  |  |  |
| 106 | *MTRNR1*:1555A>G | Homozygote |  |  |  |  |
| 107 | *MTRNR1*:1555A>G | Homozygote |  |  |  |  |
| 108 | *TMPRSS3*:A306T | Heterozygote |  |  |  |  |
| 109 | *TMPRSS3*:A306T | Heterozygote |  |  |  |  |
| 110 | *TMPRSS3*:A306T | Heterozygote |  |  |  |  |
| 111 | *TMPRSS3*:A306T | Heterozygote |  |  |  |  |
| 112 | *TMPRSS3*:A306T | Heterozygote |  |  |  |  |
| 113 | *TMPRSS3*:A306T | Heterozygote |  |  |  |  |
| 114 | *TMPRSS3*:A306T | Heterozygote |  |  |  |  |
| 115 | *TMPRSS3*:A306T | Heterozygote |  |  |  |  |
| 116 | *CDH23*:P240L | Heterozygote |  |  |  |  |
| 117 | *CDH23*:P240L | Homozygote |  |  |  |  |
| 118 | *CDH23*:P240L | Homozygote |  |  |  |  |
| 119 | *CDH23*:P240L | Heterozygote |  |  |  |  |
| 120 | *CDH23*:P240L | Heterozygote |  |  |  |  |
| 121 | *CDH23*:P240L | Heterozygote |  |  |  |  |
| 122 | *CDH23*:P240L | Heterozygote |  |  |  |  |
| 123 | *CDH23*:P240L | Heterozygote |  |  |  |  |
| 124 | *CDH23*:P240L | Heterozygote |  |  |  |  |
| 125 | *CDH23*:P240L | Heterozygote |  |  |  |  |
| 126 | *CDH23*:P240L | Heterozygote |  |  |  |  |
| 127 | *CDH23*:P240L | Heterozygote |  |  |  |  |
| **Heterozygote** | **109** | | **25** | | **1** | |
| **Homozygote** | **18** | | **0** | | **0** | |
